# Improved dietary control results in improvements in indices of white matter structure in adults with phenylketonuria: the ReDAPT study

**DOI:** 10.1017/neu.2026.10078

**Published:** 2026-04-17

**Authors:** Matthew J.Y. Kang, Christopher Adamson, Charles B. Malpas, Toby Winton-Brown, Nicholas Burgess, Tim Fazio, Dennis Velakoulis, Patricia Desmond, Vijay Venkatraman, Gerard De Jong, Mark Walterfang

**Affiliations:** 1 Neuropsychiatry Centre, The Royal Melbourne Hospital, Melbourne, Australia; 2 Department of Psychiatry, The University of Melbourne, Australia; 3 Turner Institute for Brain and Mental Health, School of Psychological Sciences, Monash University, Melbourne, Australia; 4 Department of Medicine, The Royal Melbourne Hospital, Australia; 5 Melbourne School of Psychological Sciences, The University of Melbourne, Australia; 6 Department of Psychiatry, Alfred Hospital, Melbourne, Australia; 7 Department of Neuroscience, Monash University, Australia; 8 Department of Metabolic Medicine, Royal Melbourne Hospital, Melbourne, Australia; 9 Department of Radiology, The University of Melbourne, Australia

**Keywords:** Phenylketonuria, myelination, white matter, neuroimaging, neuroprotective effects

## Abstract

**Objective:**

We tested the hypothesis that resuming dietary control in early-treated phenylketonuria (PKU) is associated with improvements in white matter integrity, using data from the ReDAPT study, which previously demonstrated cognitive and psychiatric improvements with reduced phenylalanine (Phe) levels.

**Methods:**

We re-initiated dietary control for early-treated patients with PKU and assessed the T1w/T2w ratio from standard T1-and T2-weighted magnetic resonance images, a marker of myelination and microstructural integrity. General linear mixed-effects model (GLMM) analyses were performed to assess change in the T1w/T2w ratio from baseline over twelve months after resumption of dietary control.

**Results:**

Seven participants (mean age 31 years; five female) with neuroimaging were included, with a mean of 16 years off diet and baseline Phe levels of 1157 µmol/L. GLMM analyses showed significant increases in T1w/T2w ratio over time for the whole brain (β = 0.47 [95%CI = 0.28, 0.66]), left hemisphere (β = 0.36 [95%CI = 0.19, 0.54]) and right hemisphere regions of interest (β = 0.52 [95%CI = 0.30, 0.72]). Longer time off diet was also positively associated with greater T1w/T2w changes. There was no evidence for the effects of gender or age at baseline.

**Conclusions:**

This study demonstrated significant increases in the T1w/T2w ratio in PKU patients as they resumed dietary control over a 12-month period. Raw Phe levels were not strongly associated with neuroimaging measures. These findings support the importance of lifelong treatment for PKU and also demonstrate the potential reversibility of white matter changes in the disease.

Significant outcomes

T1w/T2w ratio is a quantitative measure of brain tissue integrity, which correlated with clinical and Phe level improvements in PKU patients on dietary control.

Longer time off dietary control was associated with a greater change in T1w/T2w ratio changes.

Limitations

This was a relatively small study, limiting more nuanced exploration including the relationship between T1w/T2w ratio and PKU levels at baseline.

Highlights

We showed that improving dietary control, even modestly, improved indices of white matter integrity alongside previously demonstrated cognitive and psychiatric improvements in adult patients returning to diet after years off treatment.

## Introduction

Phenylketonuria (PKU) is a well-characterised autosomal recessive metabolic disorder where a deficiency in phenylalanine hydroxylase (PAH) activity results in elevated levels of the amino acid (AA) phenylalanine (Phe) and is the most commonly occurring inborn error of AA metabolism (van Spronsen *et al*., 2021). Heightened concentrations of Phe have been implicated in directly decreasing cerebral protein synthesis, which leads to an overall disruption in neurotransmitter activity (Hoeksma *et al*., 2009), demyelination and dendritic changes resulting in neuropsychiatric symptoms and disability in affected patients (Ashe *et al*., 2019). The current guidelines on PKU (Vockley *et al*., 2014; van Wegberg *et al*., 2017) recommend dietary control as the mainstay treatment to maintain low Phe levels whilst minimising the risk of other nutritional deficiencies. This consists of restricting natural proteins, use of Phe-free-L-AA supplements and adherence to a low protein diet. The ‘PKU diet’ has formed the cornerstone of treatment, with recommendations for lifelong adherence to dietary control in light of evidence of associations with improved neuropsychiatric symptoms (van Wegberg *et al*., 2017; Burgess *et al*., 2021).

Patients with PKU also display abnormalities on their neuroimaging, and the typical findings are T2-weighted hyperintense lesions, located in parieto-occipital regions on brain magnetic resonance imaging (MRI) (van Spronsen *et al*., 2021). More advanced methods like diffusion-weighted imaging (DWI) and diffusion tensor imaging (DTI) studies can also detect microstructural WM alterations, and few studies have investigated more advanced and research-oriented techniques including volumetric and functional neuroimaging, providing preliminary results about the impact on subcortical deep grey matter (GM) and cortical activation patterns (De Giorgi *et al*., 2023). Despite white matter (WM) abnormalities having been well documented in PKU, its exact mechanism remains unclear (Ferreira *et al*., 2021) and has been linked to a combination of myelination dysfunction, demyelination and cytotoxic damage. Furthermore, neuroimaging alterations can be incongruent with PKU-related status and clinical outcomes (Cleary *et al*., 1995; Bako & Çıkı, 2023; De Giorgi *et al*., 2023), adding to the uncertainty about their significance and limiting its use as a biomarker of treatment response to assist with informing personalised care.

There has been an interest in the use of T1w/T2w ratio (Glasser & Van Essen, 2011) to compensate for the limitations of both singular T1w and T2w images, whilst not adding additional acquisition time to an MRI protocol. T1w/T2w ratio can provide a quantitative measure of tissue integrity related to myelin content in the whole brain, by improving tissue contrast and better sensitivity related to myelin content and tissue microstructure in brain tissue. Its obvious advantage being that both T1w and T2w are conventional sequences included in most clinical MRI protocols (Ganzetti *et al*., 2014), and there is less reliance on subjective qualitative measures of WM pathology. The underlying premise is that myelin and inflammation alter the signal intensity of T1w and T2w images in opposite directions; furthermore, as T1w/T2w signal intensities are not affected by fibre orientation, it has an advantage over diffusion-based metrics in regions with crossing fibres and other complex fibre configurations. Despite the increasing interest of using T1w/T2w ratio in other neurological disorders, such as multiple sclerosis (Boaventura *et al*., 2022; Hannoun *et al*., 2022) and autoimmune encephalitis (Hartung *et al*., 2024), it has yet to be applied to PKU.

In this observational study, we aimed to explore whether the resumption of PKU diet control in previously early-treated adults improved WM abnormalities as measured by the T1w/T2w ratio, utilising an objective biomarker of neurobiological change, in a patient group previously assessed in the ReDAPT study who demonstrated improvements in cognition and psychiatric symptoms with increased dietary control (Burgess *et al*., 2021).

## Methods

### Participants

The sample included nine subjects recruited from the Metabolic Service at the Royal Melbourne Hospital, with confirmed PKU (by neonatal Guthrie card or genetic testing) who had been initiated on dietary treatment during childhood and ceased prior to 18 years of age and off diet for at least 5 years. Subjects were excluded on the basis of MRI contraindication, intellectual disability or comorbid drug or alcohol dependence. Research and ethics approval for the project was provided by the Melbourne Health Human Research Ethics Committee (MH Project Number 2014.113), and all participants provided informed consent.

### Clinical assessment

Each patient underwent a baseline assessment, with initial review by a metabolic physician and dietician for review of PKU history and diet. Serology for baseline Phe and tyrosine (Tyr) levels were taken, with repeat measures at 6 and 12-month timepoints. The cognitive proficiency index (CPI), as a measure of cognitive function, has been previously described (Burgess *et al*., 2021).

### Magnetic resonance imaging (MRI)

Each participant underwent MRI scanning on a 3T Siemens Trio TIM scanner at the Royal Melbourne Hospital.

### Calculation of the T1w/T2w ratio

FLAIR images were used for the T2w contrast and were rigidly aligned to the T1-weighted images using FLIRT [https://web.mit.edu/fsl_v5.0.10/fsl/doc/wiki/FLIRT.html]. T1w/T2w ratio calculations require calibration of the tissue-type intensities to remove inter-subject variability; the non-linear internal calibration of MRTool [www.nitrc.org/projects/mrtool] was used for this purpose (Ganzetti *et al*., 2018). This nonlinear intensity calibration is based on the intensity extracted from brain tissues (e.g. white matter, GM and cerebrospinal fluid).

**Figure 1.**
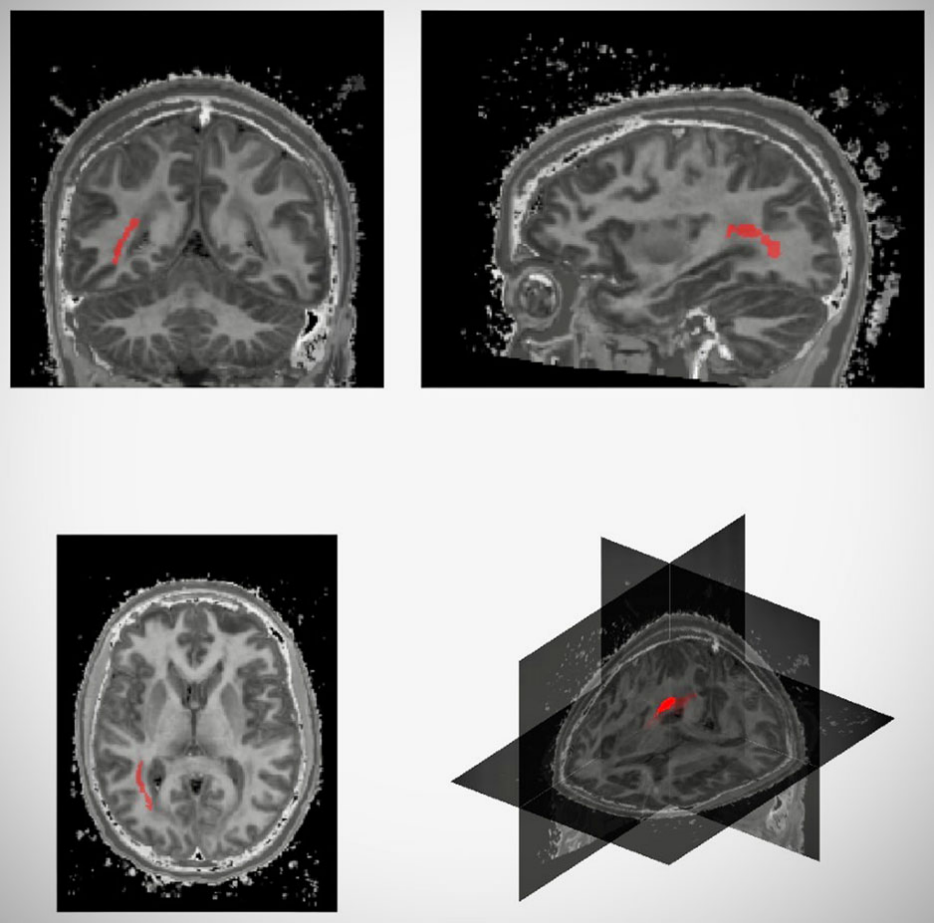
Views of the region-of-interest (ROI) in a female study subject, with coronal (top left), sagittal (top right), axial (bottom left) and cutaway views showing the right posterior region of white matter standardised across all patients and timepoints.

The region of interest (ROI) chosen for analysis (Figure 1) was the white matter region posterior to the corpus callosum in the deep white matter in each hemisphere that is commonly hyperintense on clinical images; this feature was common to all subject images. This ROI was manually marked up, using ITK-SNAP [http://www.itksnap.org/pmwiki/pmwiki.php] (Yushkevich *et al*., 2006), in the FLAIR template space image created by the ANTS software [https://github.com/PennBBL/antsjlf/blob/master/buildtemplateparallel.sh] buildtemplateparallel.sh script (Avants *et al*., 2010, 2011). Given an initial template image, which was chosen to be the average of all subject images registered to one of the subject images, it iteratively refines the template image via registration and averaging of all subject images. The left and right hemisphere ROIs were projected to each subject image via the nonlinear transformations from template to subject space. This procedure ensured the consistency of the ROIs between subjects.

### Statistical analysis

All statistical analyses were performed using the *Jamovi* software package (version 2.4.7) (Jamovi 2021), which runs on *R*. Descriptive statistics are presented as counts, means and standard deviations where appropriate. Longitudinal change in T1w/T2w ratio was investigated using general linear mixed-effects models (GLMMs) implemented using the GAMLj package (version 3.1.1) (Gallucci, 2019). ROI, the mean T1w/T2w ratio was entered as the dependent variable. Gender, time of assessment (months), years off diet and age at baseline (years) were entered as independent variables. For the secondary analyses comparing T1w/T2w ratio and clinical outcomes, we estimated longitudinal change in neuropsychiatric symptoms (CPI, HAM-A and Hamilton Depression Rating Scale (HAM-D)) adjusted for gender, years off diet and age at baseline (years). Continuous variables were scaled and centred prior to analysis. A random intercept was specified for each participant. All models were estimated using restricted maximum likelihood. Parameter estimates with 95% confidence intervals (CI) are reported for statistical inference. CIs were computed using parametric bootstrapping with 2000 replicates.

## Results

Seven of the original nine patients enrolled in the ReDAPT study (Burgess *et al*., 2021) underwent MRI scanning and are summarised in Table 1. Five participants were female, their age ranged from 19 to 47 with a mean age of 34 years. They reported that they had stopped dietary control for 19.1 years on average. The mean Phe level at baseline was 1108 µmol/L. The mean baseline CPI score derived from the (Wechsler Adult Intelligence Scale-IV scores) was 91.9. Their mean T1w/T2w ratio increased from 1.435 to 1.482 with six months of dietary treatment and to 1.508 by the end of the study. There was no correlation between blood Phe levels and T1w/T2w ratio at baseline.

**Table 1.** Sample characteristics at baseline with their serial T12/T2w ratios

*n*	Female (%)	Age (years)	Years off diet	Phe µmol/L	CPI	Baseline T1w/T2w ratio	6-month T1w/T2w ratio	12-month T1w/T2w ratio
7	5 (71)	31.1 (7.54)	16 (10.1)	1,157 (284)	89.7 (7.76)	1.435 (0.072)*	1.482 (0.066)*	1.508 (0.016)*
	Female	33	26	814	80	1.442	1.519	1.511
	Female	35	25	1,038	96	1.484	1.472	1.511
	Female	42	10	1,121	80	1.347	1.365	N/A
	Female	24	5	1,602	96	1.337	1.432	1.487
	Male	19	5	931	99	1.425	1.502	1.498
	Female	38	31	1,513	87	1.528	1.567	N/A
	Male	27	10	1,186	89	1.482	1.516	1.531

Mean (SD) unless specified.

Phe = phenylalanine level; CPI = cognitive proficiency index. NA = missing data.

* = p<0.05.

**Figure 2.**
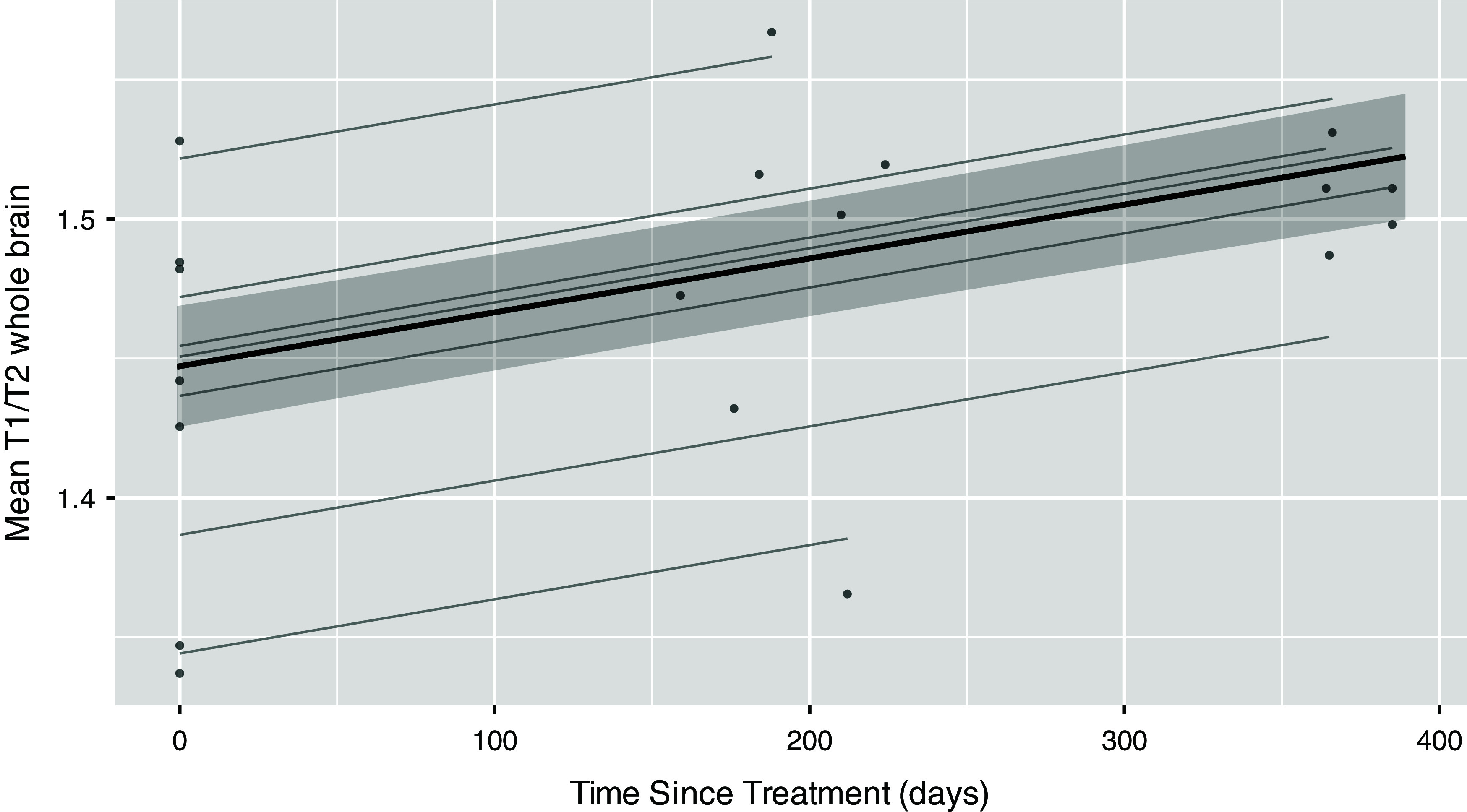
Linear mixed-effects model estimating the T1W/T2W ratio (whole brain) over time with dietary treatment.

GLMMs were used to estimate the effects of gender, age, time off diet and time from baseline on the mean T1W/T2W ratio within each ROI. As shown in Table 2, time from baseline (i.e. dietary treatment duration) was also associated with an increase in T1W/T2W ratio for the whole brain, left hemisphere and right hemisphere ROIs. In addition, more time off diet was associated with higher T1W/T2W ratio in the whole brain (figure 2), left hemisphere and right hemisphere.

**Table 2.** Model parameters

		β [95% CI]	
Parameter	Whole brain	Left hemisphere	Right hemisphere
Gender (male)	0.79 [−0.88, 2.32]	0.53 [−1.22, 2.40]	0.79 [−0.68, 2.32]
Age at baseline (years)	−0.14 [−0.96, 0.73]	−0.48 [−1.48, 0.53]	0.32 [−0.52, 1.12]
Time off diet (years)	**0.83 [0.19, 1.48]**	**0.78 [0.04, 1.52]**	**0.74 [0.14, 1.35]**
Time (months)	**0.47 [0.27, 0.65]**	**0.36 [0.19, 0.54]**	**0.52 [0.30, 0.72]**

*Note:* Values are standardised model parameters with 95% confidence intervals (CIs) derived from general linear mixed models (GLMMs). CIs were obtained from parametric bootstrapping with 2,000 replicates. Bold indicate parameters for which the CIs did not cross zero.

For the secondary analyses investigating the associations between clinical outcomes and T1w/T2w ratio (whole brain), we used GLMMs adjusted for age at baseline, gender and time off diet. Both CPI (*β* = 0.53; 95% CI [0.06, 1.00]) and HAM-A (*β* = −0.61; 95% CI [−1.16, −0.08]) were both significantly associated with the T1w/T2w ratio, whilst HAM-D (*β* = −0.51, 95% CI [−1.09, 0.05]) was not. The GLMM adjusted for age at baseline, gender and time off diet found that PhE levels were also significantly associated with T1w/T2w ratio (*β* = −1.00; 95% CI [−1.74, −0.25]).

## Discussion

Our longitudinal study found that the WM abnormality burden in patients with PKU improved with dietary control in individuals with PKU. This correlated with improvements in PhE levels and some neuropsychiatric symptoms (cognition and anxiety symptoms) as reported in our previous study (Burgess *et al*., 2021), supporting the use of T1w/T2w ratio as an objective brain-specific biomarker of dietary treatment response in PKU. The advantage of the T1w/T2w ratio is that it can be obtained from a conventional MRI brain protocol sequence and that it does not rely on subjective assessment to assess the WM disease burden.

This is the first published study to report T1w/T2w ratio in PKU, adding to the current debate (De Giorgi *et al*., 2023) about the significance of neuroimaging alterations and their relationship with neuropsychiatric symptoms. The improvement in WM abnormalities with dietary control, indicated by the increase in T1w/T2w ratio, is in line with previous studies of adults with PKU (Anderson & Leuzzi, 2010; White *et al*., 2013; Daelman *et al*., 2014). In addition, those with longer time off diet achieved greater improvements in T1w/T2w ratio with dietary control, further demonstrating the potential reversibility of WM abnormality with dietary Phe restriction treatment even in adults (Ferreira *et al*., 2021). Overall, our findings reinforce the importance of lifelong dietary control as recommended in current guidelines (Vockley *et al*., 2014; van Wegberg *et al*., 2017).

Although T1w/T2w ratio was initially proposed as a measure of myelin content (Ganzetti *et al*., 2014), its use has broadened as a marker of tissue integrity which is affected by myelin content, microstructural integrity, astrogliosis and inflammation (Boaventura *et al*., 2024). Previous research in PKU has focused on WM demyelination, but there is increasing recognition about the other impacts, including intramyelinic oedema and GM injury from neuroinflammatory processes (Anderson & Leuzzi, 2010; Ferreira *et al*., 2021; De Giorgi *et al*., 2023). Our finding of improved T1w/T2w ratios following resumption of dietary control, despite the small cohort, likely reflects that this metric captures a broader spectrum of brain integrity than myelin alone, aligning with the multifactorial effects of PhE accumulation.

We observed no correlation between baseline PhE concentrations and the T1w/T2w ratio. This finding contrasts with previous studies using larger sample sizes and established neuroimaging methods, which have consistently demonstrated associations between Phe levels and white matter abnormalities (Antenor-Dorsey *et al*., 2013; White *et al*., 2013; Hood *et al*., 2015; González *et al*., 2018; Clocksin *et al*., 2021). Given our small sample size, this null finding most likely reflects insufficient statistical power to detect between-subjects effects.

In contrast, improvements in the T1w/T2w ratio did correspond with reductions in Phe over time within subjects. While speculative, one possible explanation for the discordance between our cross-sectional and longitudinal findings is that the T1w/T2w ratio may be particularly sensitive to changes in the cumulative burden of hyperphenylalaninaemia, encompassing both the magnitude and duration of exposure, which may be better captured through within-subject longitudinal assessment than cross-sectional comparison in a small cohort. This interpretation would also be consistent with the individual variation in MRI appearance and Phe levels cross-sectionally noted in existing literature (Cleary *et al*., 1995; White *et al*., 2010). This hypothesis should be interpreted cautiously and requires confirmation in larger, adequately powered studies that include both cross-sectional and longitudinal assessments.

Nevertheless, the requirement for MRI scanning limits broad clinical applicability due to cost, accessibility and patient burden. Emerging blood-based biomarkers to monitor neurological disease progression like glial fibrillary acidic protein and neurofilament light chain, may offer a practical and scalable adjunct to neuroimaging (Lotz-Havla *et al*., 2022; Cawley *et al*., 2025; Eratne *et al*., 2025). Combining MRI-derived indices like the T1w/T2w ratio with circulating biomarkers could facilitate multimodal frameworks for monitoring brain health in PKU, where MRI serves as a confirmatory or mechanistic reference, and blood tests provide an accessible means for ongoing assessment.

This study was limited by a small sample size, thus requires replication on a larger scale. The use of CIs allowed us to understand the effect that the small sample size had on parameter uncertainty, which was relatively minimal. Furthermore, our data were acquired using the same MRI protocol and sequence parameters, which is important for more robust and homogeneous results. Future research could explore the potential of combining T1w/T2w ratio with other biomarkers, such as neuroinflammatory markers or cerebrospinal fluid analysis, to provide a more comprehensive understanding of brain integrity in PKU. Replicating this study in a more diverse population, including varying ages, genders and ethnic backgrounds, would help generalise these findings and determine whether certain subgroups benefit more from specific treatment aimed at lowering Phe.

## Conclusions

Our study demonstrated that early-treated patients who resumed dietary control of PKU had improvements in their T1w/T2w ratio. This ratio, which can be obtained through standard MRI protocols, offers a reliable, non-invasive and objective marker for assessing brain integrity and WM burden in response to dietary treatment. The findings underscore the importance of lifelong adherence to dietary management in PKU, as the positive effects on WM abnormalities and potential reversal of damage are particularly evident in individuals with extended periods off diet. Additionally, the study highlights the broader utility of the T1w/T2w ratio as a marker of overall brain integrity, potentially extending its application beyond PKU to other neurological conditions. While our results are promising, they require further validation in larger, more diverse cohorts. Nonetheless, these findings contribute valuable insights into the role of dietary control in mitigating the neurological impact of PKU and emphasise the necessity for continuous monitoring and personalised treatment approaches in managing this condition.
